# Retraction: Effect of Adiponectin on Kidney Crystal Formation in Metabolic Syndrome Model Mice via Inhibition of Inflammation and Apoptosis

**DOI:** 10.1371/journal.pone.0314521

**Published:** 2024-11-21

**Authors:** The PLOS ONE Editors

Following the publication of this article [1], concerns were raised regarding the western blot results in Fig 6. Specifically,

- The following bands appear similar when aspect ratios are adjusted:
  ○ LYZ1 lane 1 and STAT3 lanes 1 and 3 flipped horizontally
  ○ LYZ1 lane 3 and STAT3 lane 5 flipped horizontally
  ○ LYZ1 lane 4 and STAT3 lane 4
  ○ LYZ1 lane 5 and STAT3 lane 2
- There appear to be vertical discontinuities in the background of the following panels suggestive of splicing: OPN, MCP-1, LYZ1 and STAT3 panels.
- The β-actin panels in the left and right columns appear similar.

The corresponding author stated that the original data underlying the results in this figure are no longer available as the data were disposed of after the retention period required by institutional regulations. The Editors regret that the concerns regarding these figures were not discussed with the authors before the disposal of the data.

The corresponding author acknowledged that the indicated bands in the LYZ1 and STAT3 panels appear similar, and stated that in the absence of original data, they are unable to answer the concerns.

The corresponding author provided data from a repeat of the western blot experiment presented in Fig 6. The Editors do not consider the repeat data to be sufficient to fully support the published results.

The *PLOS ONE* Editors retract this article due to unresolved concerns which call into question the integrity and reliability of the results.

All authors did not agree with the retraction.
